# Urban poverty and nutrition challenges associated with accessibility to a healthy diet: a global systematic literature review

**DOI:** 10.1186/s12939-020-01330-0

**Published:** 2021-01-20

**Authors:** Mireya Vilar-Compte, Soraya Burrola-Méndez, Annel Lozano-Marrufo, Isabel Ferré-Eguiluz, Diana Flores, Pablo Gaitán-Rossi, Graciela Teruel, Rafael Pérez-Escamilla

**Affiliations:** 1 Research Institute for Equitable Development EQUIDE, Universidad Iberoamericana, Prolongación Paseo de Reforma 880, Lomas de Santa Fé, 01219 Mexico City, Mexico; 2 Yale School of Public Health, 60 College Street, New Haven, CT 06510 USA

**Keywords:** Urban poverty, Nutrition, Social protection, Public health, Food security

## Abstract

**Background:**

There is an increasing global trend towards urbanization. In general, there are less food access issues in urban than rural areas, but this “urban advantage” does not benefit the poorest who face disproportionate barriers to accessing healthy food and have an increased risk of malnutrition.

**Objectives:**

This systematic literature review aimed to assess urban poverty as a determinant of access to a healthy diet, and to examine the contribution of urban poverty to the nutritional status of individuals.

**Methods:**

Following the Preferred Reporting Items for Systematic Reviews and Meta-Analysis (PRISMA) methodology, our review included quantitative and qualitative studies published in English or in Spanish between 2000 and 2019. The articles were eligible if they focused on nutrition access (i.e. access to a healthy diet) or nutrition outcomes (i.e., anemia, overweight and obesity, micronutrient deficiency, micronutrient malnutrition) among urban poor populations. Articles were excluded if they did not meet pre-established criteria. The quality of the quantitative studies was assessed by applying Khan et al.’s methodology. Similarly, we assessed the quality of qualitative articles through an adapted version of the National Institute for Health and Care Excellence (NICE) methodology checklist. Finally, we systematically analyzed all papers that met the inclusion criteria based on a qualitative content and thematic analysis.

**Results:**

Of the 68 papers included in the systematic review, 55 used quantitative and 13 used qualitative methods. Through the analysis of the literature we found four key themes: (i) elements that affect access to healthy eating in individuals in urban poverty, (ii) food insecurity and urban poverty, (iii) risk factors for the nutritional status of urban poor and (iv) coping strategies to limited access to food. Based on the systematization of the literature on these themes, we then proposed a conceptual framework of urban poverty and nutrition.

**Conclusions:**

This systematic review identified distinct barriers posed by urban poverty in accessing healthy diets and its association with poorer nutrition outcomes, hence, questioning the “urban advantage”. A conceptual framework emerging from the existing literature is proposed to guide future studies and policies.

**Systematic review registration:**

PROSPERO Registration number: CRD42018089788.

**Supplementary Information:**

The online version contains supplementary material available at 10.1186/s12939-020-01330-0.

## Background

Urbanization is a rising global phenomenon. Today 55% of the global population lives in urban areas, and it is estimated that by 2050 70% of the population will live in one of them [1]. Compared to rural areas, urban regions feature greater social and economic development, more labor opportunities, and access to more diverse and better essential services. However, urban areas also concentrate poverty [2]. The urban poor not only lack income and resources to ensure an adequate wellbeing, but frequently experience limited access to basic services, labor opportunities and to possibilities for social development. Prior studies highlight increasing trends in urban poverty, partially resulting from accelerating urbanization processes in low-and middle-income countries; it has been estimated that by 2035 the majority of individuals in extreme poverty (i.e. daily income less than US1.25) will live in urban areas [1, 3].

These challenges have been addressed in the Sustainable Development Goals (SDG) [4]; specifically, SDG 11 establishes that countries need to have urban sustainable development plans to promote the wellbeing of people, especially the most socioeconomic vulnerable. Furthermore, SDG 1 states that all forms of poverty should be eradicated by 2030.

The SDGs are also strongly linked with food insecurity (FI) [5]. Urban environments imply a particular risk for FI and poor nutrition outcomes since access to food depends on the commercial supply that, in turn, is linked to income levels [6, 7]. On the one hand, it has been previously recognized that the urban poor are particularly vulnerable to macroeconomic shocks that affect their capacity to generate income which in turn leads them to consume less healthy diets [8, 9]. On the other hand, previous studies suggest that urban diets, on average, are better than rural diets because they are more diverse and, given the food distribution systems, there is greater access to products such as animal proteins [10]. However, this supposed urban advantage is not equally distributed as it does not extend to the poorest socioeconomic strata.

Previous research indicates that there are geographic differentials in access to food [11], which are linked to economic barriers in accessing healthy food options [12]. Hence, those with lower incomes do not have access to diets rich in heathy foods including fresh fruits and vegetables, tubers, and legumes. Instead they have relatively more access and consume higher amounts of sugars, fats, and highly processed or ultra-processed foods [13]. Although this phenomenon has been generically identified as part of the “nutritional transition”, it is important to emphasize that in urban centers, these outcomes are linked to socioeconomic inequities [6]. Ultra-processed products have a high energy density, have long shelf lives, many are ready-to-eat and they are relatively cheaper [14, 15]. All these features make them convenient for urban and low-income individuals who may have limited resources such as household heating and cooking goods, safe drinking water supply, and sanitation, amongst other basic needs. A study of 74 countries from the Pan-American Health Organization conducted in 2015 found that sales of ultra-processed products were larger in more urbanized countries, and that the market is expanding to poorer sectors [16].

Food environments can influence the risk of malnutrition and corresponding infectious and non-communicable chronic diseases. In urban areas, food deserts and food swamps – understood as regions with very limited or difficult access to supermarkets and healthful food choices [17] – exemplify challenging food environments, which are generally more common in low-income urban areas [18]. These environments are in turn associated with unequal nutrition outcomes. For example, in Latin America, the risk of chronic malnutrition in urban children under 5 years of age is ten times higher among the poorest compared with their counterparts falling in the highest socioeconomic level [7].

Despite such compelling evidence, there are few studies that have attempted to document in detail the food access challenges and their relationship with different nutritional outcomes among poor urban populations. Therefore, the aim of this study was to conduct, from a global perspective, a systematic literature review (SLR) to assess urban poverty as a determinant of access to a healthy diet, and to document the association between urban poverty and the nutritional status of individuals.

## Methods

The protocol for this systematic review was registered on PROSPERO prior to starting the literature search (CRD42018089788).

The review centered in nutrition outcomes related to: (i) access to a healthy diet as defined by the World Health Organization [19], which includes aspects of variety, quantity, balance and food safety, and (ii) nutrition outcomes related to the SDGs – anemia, overweight and obesity, micronutrient deficiency, and micronutrient malnutrition [20]. These outcomes were kept generic and subsequently categorized through the operationalizations used in the studies. The exposure variable of interest was urban poverty. Poverty was captured through different indicators such as income thresholds, poverty lines, multidimensional poverty measures, socioeconomic indexes (based on assets and services), wealth indexes, geographic areas considered highly vulnerable or lacking basic services (i.e. slums), or people participating in social programs targeted at the vulnerable/low income. Similarly, “urban” as a context where poverty happens was not defined through a unique criterion – as different countries used different criteria. Hence, “urban” was defined in terms of population size, population density, type of economic activity, level of infrastructure, or a combination of these criteria.

### Inclusion and exclusion criteria

This systematic review followed the guidance of the Preferred Reporting Items for Systematic Reviews and Meta-Analyses (PRISMA) [21, 22]. We prepared a literature search protocol to define a priori inclusion criteria (see Table 1). Qualitative and quantitative studies were included if they focused on nutrition access or nutrition outcomes among urban poor populations (i.e. individuals, families, households). Quantitative studies could be observational or experimental.

**Table 1:** Inclusion criteria for urban poverty and nutrition studies

Criteria	Inclusion
Type of Literature	Peer reviewed journal articles.
Type of Studies	Qualitative or quantitative empirical studies.
“Intervention”	Studies looking at individuals or households described as poor through income, assets, geographic location/areas lacking basic services, participation in social program for socially disadvantaged groups or those directly defined as poor through specific poverty indexes.
Level of Analysis	Analyses of poor individuals, families or households settled in urban areas.
Analytical Perspective	Descriptive analyses or in-depth cases looking at the urban poor. Comparative analyses comparing urban poor with urban non-poor or with rural poor.
Outcome	Healthy diet, anemia, overweight and obesity, micronutrient deficiency, micronutrient malnutrition.
Target Population	Urban populations. “Urban” could be defined in terms of population size, population density, type of economic activity, level of infrastructure, or a combination of these or other criteria.

Studies were excluded if they focused on the general population (i.e. without a specific focus on urban and poor settings) or if they were centered in populations with special conditions (i.e. refugees, prisoners). Only peer reviewed studies published in English or Spanish were included in the review.

### Search strategy

Four bibliographical databases (PubMed, Web of Science, Scielo and EBSCO) were systematically searched for studies published between January 2000 and January 2019. The year 2000 was selected as a threshold because urbanization was recognized as key in the Millennium Development Goals (MDGs) linked to poverty and the health outcomes of individuals. Indeed, the MDGs led to specific research and interventions targeting the urban vulnerable populations [23–30]. Relevant literature was identified following the Boolean search algorithms summarized in Supplementary Table 1. Free-text terms were used to generate search strategies for each database. Studies identified through each database were imported to Excel, and then duplicates were identified and removed. The studies were then imported to EndNote [31].

### Study selection

In the first phase, abstracts were reviewed by three of the authors (DF, IF and SB) who were standardized to screen titles and abstracts of studies identified in the search. Articles were excluded if they did not meet the criteria established in Table 1. They were included if there was an indication that access to healthful foods or any of the nutrition outcomes of interest were being described or analyzed, either through qualitative or quantitative approaches, in urban poor/vulnerable populations. In the next phase, articles were retrieved and independently assessed for eligibility (see criteria in Table 1). Consensus was reached in consultation with a fourth author (MVC) as needed.

### Data extraction

The following information was extracted from each study: (i) methods (i.e. qualitative or quantitative study design, and corresponding details); (ii) territorial definition of the urban space (i.e. urban or semi-urban, large cities, slums, etc.); (iii) poverty definition; and (iv) operationalization of the food and nutrition variables (i.e. food access, nutrition outcomes). In addition, data were extracted to describe the study sample, confounding or mediating factors, statistical tests or data triangulation, and key findings.

### Quality assessment

The studies’ quality assessment was conducted by reviewing each study according to specific guidelines. For quantitative studies, guidelines were adapted from those proposed by Khan [22] which focus on four aspects: (i) type of design; (ii) how exposure was operationalized; (iii) how outcome variables were ascertained; and (iv) if confounding variables were controlled for. Supplementary Table 2 provides further details on the definition of each of these elements. For qualitative studies a guideline was adapted from the National Institute for Health and Care Excellence (NICE) methodology checklist for qualitative studies [32]. Five quality domains were assessed for each study: (i) theoretical approach; (ii) study design; (iii) data collection; (iv) validity; and (v) analysis. Supplementary Table 3 defines how each of the areas were specifically assessed. Quality assessment was performed by two researchers (SB, IF); when there were conflicting results a third reviewer (ALM, MVC) provided input until consensus was reached. To estimate the agreement between reviewers, a Cohen’s Kappa statistic was computed.

### Analysis of the systematized papers

The purpose of systematically examining the studies was to generate a common understanding about how urban poverty shapes nutrition (both in terms of access and outcomes). The analysis of the studies was based on a qualitative content and thematic analyses. The objective of such perspective was to analyze the textual data from the studies to elucidate themes [33]. Hence, a three folded analytical process was followed: (i) data from the studies was coded in NVivo 12 [34]; nodes were generated and significant information from the systematized papers was dropped in such nodes; (ii) meaning of the information in the different nodes was examined; and (iii) themes were generated. This analysis was performed by three of the authors (MVC, DF, SB) based on consensus about the nodes, meanings and themes. These findings led to proposing a conceptual framework about how urban poverty shapes nutrition.

## Results

### Description of the studies

**Fig. 1:**
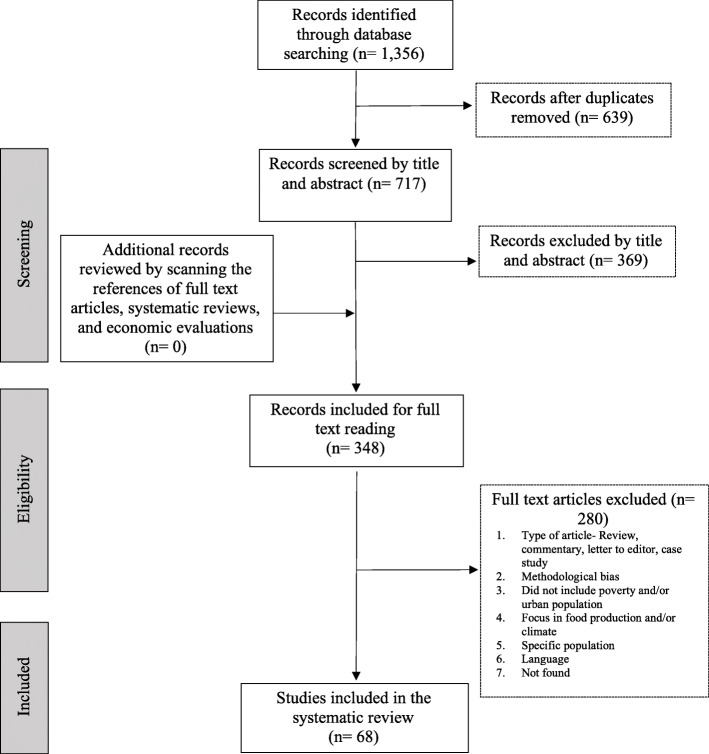
Preferred Reporting Items for Systematic Reviews and Meta-Analyses (PRISMA) Diagram

Figure 1 follows the PRISMA structure [22] and provides a detailed summary of the research results. After duplicated studies were removed, the abstracts of 717 records were screened, leading to 348 papers for full review. Sixty-eight studies met the eligibility criteria and quality assessment and were included in the review. Among these studies, the majority (81%) used quantitative methods, while fewer focused on qualitative approaches (19%). The average Cohen’s Kappa statistic between-reviewers for quantitative studies was 0.963 (an almost perfect agreement), and for qualitative studies 0.759 (a substantial level of agreement) [35].

The geographical distribution of the included studies is presented in Table 2. Based on the categorization by regions as classified by the World Bank [36], close to two thirds of the papers were based on studies conducted in the Americas (i.e. 39.7% in North America and 25% in Latin American & Caribbean), followed by 17.6% in Sub-Saharan Africa, and 17.6% in East Asia & Pacific. Only 8.8% were from South Asia, 5.9% from Europe & Central Asia, and 2.9% from Middle East & North Africa.

**Table 2:** Geographic distribution of the reviewed papers, operationalization of poverty and the urban space

Region being studied^a^	Total^b^	%^b^
Sub-Saharan Africa	12	17.6
North America	27	39.7
Latin America & Caribbean	17	25.0
South Asia	6	8.8
East Asia & Pacific	12	17.6
Europe & Central Asia	4	5.9
Middle East & North Africa	2	2.9

^a^The categorization by regions and income level of the country corresponds to the World Bank classification (2020)

^b^The percentage corresponds to the total by category among the 68 articles reviewed

Tables 3 and 4 provide information on how studies operationalized the poverty construct. It was commonly defined through mainstream economic classifications such as: lower deciles or quintiles of income distribution (18.9%); low socioeconomic level, ascertained through education level, type of employment, or social class (17.6%); poverty lines or thresholds based on a minimum income to satisfy basic needs, or through more complex multidimensional measures of poverty (13.5%); composite measures such as assets indexes (5.4%) or social vulnerability indexes (2.7%); and relative household’s expenditure measures (1.4%) – which are commonly used in the economics literature due to their strong theoretical background. Together, these definitions of poverty or vulnerability were used in more than half of the studies (59.5%).

**Table 3:** Quality assessment guidelines for quantitative studies

Author (year)	Country (City)	Design	Specific population	Definition urban space	Definition poverty/ vulnerability	Comparison group	Access measure	Nutritional outcomes	Controls for confounders
Agarwal et al. (2009) [37]	India (Delhi)	Cross-sectional	Households	Slum	Household assets wealth index	Urban poor without food insecurity	Food security	✘	✓
Akinboade & Adeyefa (2018) [38]	South Africa (Tshwane)	Cross-sectional	Households	Area within a city	Low income level	✘	Food security	✘	✘
Appelhans, Waring, Schneider, & Pagoto (2014) [39]	United States (Chicago)	Cross-sectional	Households	City	Poverty line/ Poverty threshold	✘	Child dietary pattern	✘	✓
Azambuja, Netto-Oliveira, Oliveira, Azambuja, & Rinaldi (2013) [40]	Brazil (Cruzeiro do Oeste)	Cross-sectional	Children 6 to 10 years old	City	Low socioeconomic level	Urban non-poor	✘	Overweight; obesity	✓
Barosh, Friel, Engelhardt, & Chan (2014) [41]	Australia (Sydney)	Cross-sectional	Food retail stores	Area within a city	Area defined as poor; low income level	Urban non-poor	Food basket affordability	✘	✘
Battersby & Peyton (2014) [42]	South Africa (Cape Town)	Geospatial	Food retail stores	City	Low income level	✘	Distribution of food retail stores	✘	✘
Battersby (2019) [43]	Multi-site	Cross-sectional	Households	City	Area defined as poor	✘	Food security	✘	✘
Belachew et al. (2012) [44]	Ethiopia (Jimma zone)	Longitudinal/Panel	Adolescents 13 to 17 years old	City	Low income level	Urban non-poor; semiurban poor; rural poor	Food security	✘	✓
Birhane, Shiferaw, Hagos, & Mohindra (2014) [45]	Ethiopia (Addis Ababa)	Cross-sectional	Households	City	Low income level	Urban non-poor	Food security; dietary diversity	✘	✓
de Souza Bittencourt, Chaves dos Santos, de Jesus Pinto, Aliaga, & de Cássia Ribeiro-Silva (2013) [46]	Brazil (Salvador)	Cross-sectional	Children 6 to 12 years old	Area within the city	Poverty line/ poverty threshold	Urban poor with different levels of food insecurity	Food security	✘	✓
Castañeda-Castaneira, Ortiz-Pérez, Robles-Pinto, & Molina-Frechero (2016) [47]	Mexico (Mexico City)	Cross-sectional	Children from 6 to 11 years old	Area within a city	Low socioeconomic level	✘	Child dietary pattern	✘	✘
Cavanagh, Jurkowski, Bozlak, Hastings, & Klein (2017) [48]	United States (New York City)	Case-control	Patients with obesity, hypertension and/or diabetes.	Area within a city	Poor area	Matched controls not receiving the program	✘	Body mass index	✘
Chambers, Duarte, & Yang (2009) [49]	United States (Multi-site)	Cross-sectional	Dyad Mother-Child	City	Poor area	Urban poor with different levels of household instability (psychosocial and economic constraints)	✘	Obesity	✓
Costa, Horta, & Ramos (2019) [50]	Brazil (Belo Horizonte)	Cross-sectional	Restaurant workers	City	Low income level	Urban non-poor	Food security	Overweight; obesity	✓
Cunha, Sichieri, de Almeida, & Pereira (2011) [51]	Brazil (Rio de Janeiro)	Cross-sectional	Adults 19 to 65 years old	Area within the city	Low income level	Urban poor with different dietary patterns	Dietary pattern	✘	✓
Davies, Frausin, & Parry (2017) [52]	Brazil (Multi-site)	Geospatial	Cities within the Brazilian Amazon	City	Poor area	✘	Food retail stores	✘	✘
Faye, Baschieri, Falkingham, & Muindi (2011) [53]	Kenya (Nairobi)	Longitudinal/ Panel	Households	Slums	Low income level	Urban non-poor	Food security	✘	✓
Floro & Bali Swain (2013) [54]	Multi-site	Cross-sectional	Household	Slums	Poor area	Urban poor with different types of enterprises	Food security	✘	✓
Garcia et al. (2018) [55]	United States (Baltimore)	Cross-sectional	Adult caregiver of 4 to 19-year-old children	City	Low income level	Urban non-poor	Dietary pattern	✘	✓
Gundersen, Lohman, Eisenmann, Garasky, & Stewart (2008) [56]	United States (Multi-site)	Cross-sectional	Children 10 to 15 years old	City	Low income level	Urban non-poor, urban food secure	✘	Overweight	✓
Jones & Charlton (2015) [57]	Republic of Vanuatu (Port Vila)	Cross-sectional	Household	City	Relative household expenditure	Urban non-poor	Fruit and vegetable affordability	✘	✘
Kasper, Gupta, Tran, Cook, & Meyers (2000) [58]	United States (Multi-site)	Cross-sectional	Legal immigrants	City	Poverty line/ poverty threshold	Urban food secure	Food security	✘	✘
Kirkpatrick & Tarasuk (2011) [59]	Canada (Toronto)	Cross-sectional	Households	City	Poverty line/ poverty threshold	Urban non-poor	Food security	✘	✓
Lim, Zoellner, Ajrouch, & Ismail (2011) [60]	United States (Detroit)	Cohort	Adult caregiver of 3 to 5-year-old children	City	Poor area	Urban non-poor	✘	Change in weight category over time	✓
Lohman, Stewart, Gundersen, Garasky, & Eisenmann (2009) [61]	United States (Multi-site)	Cross-sectional	Dyad caregiver- adolescents 10 to 15 years old	City	Poor area and Poverty line/ poverty threshold	Urban non-poor	✘	Overweight; obesity	✓
Lopes, Sichieri, Salles-Costa, Veiga, & Pereira (2013) [62]	Brazil (Rio de Janeiro)	Cross-sectional	Adolescents 12 to 18 years old	City	Low income level	Urban non-poor	Dietary patterns	Overweight; stunting	✓
Manyanga et al. (2017) [63]	Multi-site	Cross-sectional	Children 9 to 11-year-old	City	Low socioeconomic level	Urban non-poor	Dietary patterns	✘	✓
Martinez, Clark, & Gudzune (2019) [64]	United States (Baltimore)	Cross-sectional	Households	City	Housing condition	Urban without access to a vehicle	Food security; dietary pattern	✘	✓
Martin-Fernandez, Grillo, Parizot, Caillavet, & Chauvin (2013) [65]	France (Paris)	Cross-sectional	Adults	Metropolitan area	Poverty line/ poverty threshold	Urban non-poor	Food security	✘	✓
McCordic & Abrahamo (2019) [66]	Mozambique (Multi-site)	Cross-sectional	Households	City	Household assets wealth index	Urban with different levels of food security	Food security	✘	✓
Miller et al. (2008) [67]	United States (Chelsea)	Cross-sectional	Households	City	Food security	Urban food secure	✘	Overweight; obesity; underweight; anemia; high lead	✘
Morton, Bitto, Oakland, & Sand (2008) [68]	United States (Multi-site)	Cross-sectional	Adults	Area within a city	Poor area	Rural poor	Coping strategies for food access	✘	✘
Mutisya, Kandala, Ngware, & Kabiru (2015) [69]	Kenya (Nairobi)	Cohort	Dyad mother-Child 6 to 24 months old	Slum	Household assets wealth index	Urban with different levels of food security; urban with different levels of poverty	✘	Stunting	✓
Nascimento-Ferreira et al. (2014) [70]	Brazil (Imperatriz)	Cross-sectional	Adolescent 14 to19 years old	City	Low socioeconomic level	Urban non-poor	✘	Abdominal obesity; overweight; obesity; high blood pressure	✓
Odunitan-Wayas et al. (2018) [71]	South Africa (Cape Town)	Cross-sectional	Supermarket costumers	City	Poor area; Food security	Urban non-poor; Urban food secure	Purchase pattern; access to food retail stores; food environment perception	✘	✓
Omidvar, Ghazi-Tabatabie, Sadeghi, Mohammadi, & Abbasi-Shavazi (2013) [72]	Iran (Multi-site)	Cross-sectional	Migrant women	City	Low socioeconomic level	Urban non-poor	Food security	✘	✓
Ortiz-Hernández, Acosta-Gutiérrez, Núñez-Pérez, Peralta-Fonseca, & Ruiz-Gómez (2007) [73]	Mexico (Mexico City)	Cross-sectional	Elementary school students 4° to 6° grade	Area within a city	Low socioeconomic level	Urban non-poor	Dietary patterns	Obesity	✓
Park et al. (2011) [74]	United States (New York City)	Cross-sectional	Female caregivers	Area within a city	Poor area	Urban non-poor	Dietary patterns	✘	✓
Ponce, Ramirez, & Delisle (2006) [75]	Mexico (Multi-site)	Cross-sectional	Men 35 to 65 years old	City	Poor area	Urban non-poor; rural poor	Dietary diversity; Micronutrient adequacy score	✘	✓
Ramsey, Giskes, Turrell, & Gallegos (2012) [76]	Australia (Brisbane)	Cross-sectional	Adults 25 to 45 years old	Area within a city	Low income level	Urban non-poor	Food security	✘	✓
Rani et al. (2018) [77]	India (Varanasi)	Cross-sectional	Female adolescents	Slums	Low income level	✘	✘	✘	✓
Russell & Heidkamp (2011) [78]	United States (New Haven)	Geospatial	Food retail stores	City	Poor area	Urban non-poor.	Distribution of food retail stores	✘	✘
Sarki, Robertson, & Parlesak (2016) [79]	Nepal (Lalitpur)	Cross-sectional	Dyad mother-child 0 to 5 years old	Area within a city	Low socioeconomic level	Urban non-poor	✘	Stunting; underweight; overweight; obesity	✘
Shaw (2012) [80]	England (Birmingham)	Geospatial	Food retail stores that sell fresh produce	City	Low socioeconomic level	Urban non-poor	Distribution of food retail stores selling fresh produce	✘	✘
Tsai & Rosenheck (2013) [81]	United States (Multi-site)	Cross-sectional	Homeless	City	Homelessness	✘	✘	Overweight; obesity	✘
Vedovato et al. (2016) [82]	United States (Baltimore City)	Cross-sectional	Households	City	Poor area	Urban with different levels of food security	Food security	Overweight; obesity	✓
Villamor et al. (2017) [83]	Multi-site	Cross-sectional	Households	Peri-urban area	Low socioeconomic level	Urban non-poor	✘	Metabolic syndrome	✓
Vuong, Gallegos, & Ramsey (2015) [84]	Vietnam (Ho Chi Minh)	Cross-sectional	Household	Area within a city	Poverty line/ poverty threshold; Household assets wealth index	Urban non-poor	Food security	✘	✓
Wagner et al. (2019) [85]	Multi-site	Cross-sectional	Households	Metropolitan area	Social vulnerability index; food security	Urban non-poor; urban with different levels of food security	Dietary pattern; household food sources	✘	✘
Wang et al. (2012) [86]	China (Multi-site)	Cross-sectional	Adults 18 to 74 years old	Area within a city	Low socioeconomic level	Urban non-poor	✘	Abdominal obesity; overweight; obesity	✓
Whitaker & Orzol (2006) [87]	United States (Multi-site)	Cross-sectional	3-year-old children	City	Poverty line/ poverty threshold	Urban non-poor.	✘	Obesity	✓
Wrathall (2014) [88]	Multi-site	Longitudinal/ Panel	Country	Slums	Poor area	✘	✘	Obesity; body mass index	✓
Yaemsiri, Olson, He, & Kerker (2012) [89]	United States (New York City)	Cross-sectional	Adults	Area within a city	Poverty line/ poverty threshold	✘	Food security	✘	✓
Zhai, Xue, Luo, Zhang, & Cheng (2018) [90]	China (Chengdu)	Cross-sectional	Children 7 to 12 years old	City	Low socioeconomic level	Urban non-poor	✘	Overweight; obesity	✓
Zhang & Debarchana (2016) [91]	United States (Hartford)	Geospatial	Food retail stores	City	Social vulnerability index	Urban non-poor	Distribution of food retail stores	✘	✘

**Table 4:** Quality assessment guidelines for qualitative studies

Author, year	Country (City)	Theoretical approach		Study design		Data collection	Validity		Analysis			Specific population	Definition of urban space	Definition of poverty/ vulnerability
		Appropriate approach	Clear aims	Adequate plan	Adequate sample & sampling	Definition of data collection, recording, transcription	Description of context, participants, biases	Triangulation	Richness of data	Coding process described	Findings are supported			
Chan, DeMelo, Gingras, & Gucciardi (2015) [92]	Canada (Toronto)	✓	✓	✓	✓	✓	✓	✓	✓	✓	✓	Adults with diabetes	Area within a city	Low income level; food security
Cotter, Teixeira, Bontrager, Horton, & Soriano (2017) [93]	United States (Washington)	✓	✓	✓	✓	✓	✓	✓	✓	✓	✓	Adults	Area within a city	Low socioeconomic level
Crawford et al. (2014) [94]	Australia (Sydney)	✓	✓	✓	✓	✓	✓	✓	✓	✓	✓	Homeless 15 to 25 years old	Metropolitan area	Homelessness
Dachner & Tarasuk (2002) [95]	Canada (Toronto)	✓	✓	✓	✓	✓	✓	✓	✓	✓	✓	Homeless youth above 16 years old	Area within a city	Homelessness
de Morais Sato et al. (2017) [96]	Brazil (Santos)	✓	✓	✓	✓	✓	✓	✓	✓	✓	✓	Adult mothers	Area within a city	Poor area
Dubowitz et al. (2007) [97]	United States (Multi-site)	✓	✓	✓	✓	✓	✓	✘	✓	✓	✓	Mothers	Metropolitan area	Poverty line/ poverty threshold
Green-LaPierre et al. (2012) [98]	Canada (Nova Scotia)	✓	✓	✓	✓	✓	✓	✓	✓	✓	✓	Women 65 years of age or older	City	Low socioeconomic level
Hammelman (2018) [99]	United States (Washington)	✓	✓	✓	✓	✓	✓	✘	✓	✘	✓	Migrant women	Area within a city	Food security
Leung et al. (2016) [100]	United States (New York City)	✓	✓	✓	✓	✓	✓	✓	✓	✓	✓	Youth 11 to 14 years old	Area within a city	Poor area
Levay, Mumtaz, Faiz Rashid, & Willows (2013) [101]	Bangladesh (Dhaka)	✓	✓	✓	✓	✓	✓	✓	✓	✓	✓	Pregnant women and new mothers	Slum	Poor area
McInvale Trejo & Shaw-Ridley (2019) [102]	Peru (Lima)	✓	✓	✓	✓	✓	✓	✓	✓	✓	✓	Dyad parent-child 3 to 4 years-old	Area within the city	Poor area
Piaseu, Belza, & Shell-Duncan (2004) [103]	Thailand (Bangkok)	✓	✓	✓	✓	✓	✓	✓	✓	✘	✓	Women 25 to 60 years old	Slum	Poor area
Wicks, Trevena, & Quine (2006) [104]	Australia (Sydney)	✓	✓	✓	✓	✓	✓	✓	✘	✓	✓	Homeless and marginally housed adults	Area within a city	Food security

The second most common metrics used for determining poverty status was through geographical characteristics (27%). Based on community, municipality or other geographic units, the studies defined the poverty status based on access to services or gradients of human development, among others. The degree of specification of how “poor areas” were defined varied across studies. Finally, another subset of the studies included in the SLR defined poverty and vulnerability through specific unidimensional conditions such as poor housing conditions, FI or homelessness (13.5%).

Tables 3 and 4 also provide information about how the “urban” space was ascertained in the studies. More than half of the studies (54.4%) defined broadly the urban space as “cities” or “metropolitan areas”. Around one third of the studies (32.4%) centered in areas within a city, while 13.3% of the studies focused in specific peri-urban areas or slums.

**Fig. 2:**
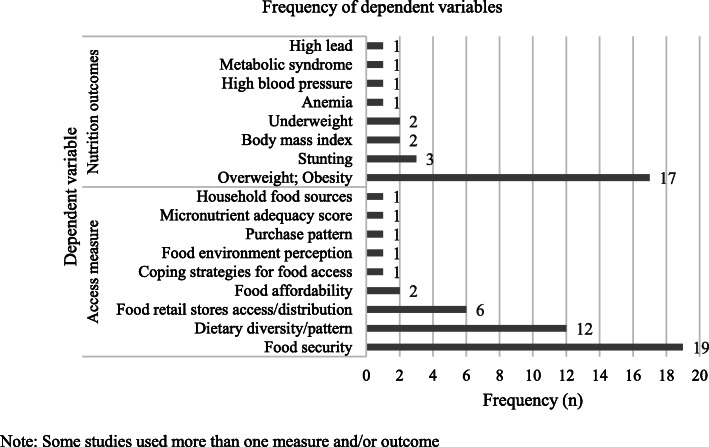
Access measures and nutrition outcomes used as dependent variables in quantitative studies. Note: Some studies used more than one measure and/or outcome

Among the quantitative studies (*n* = 55), 63% analyzed food access measures as dependent variables, 30% as nutrition outcomes, and 7% as both. As portrayed in Fig. 2, the most common operationalization of access was through food security scales, dietary diversity indexes or scores, and through assessments of access to retail food stores. On the other hand, overweight and obesity and stunting were the most commonly assessed nutrition outcomes. Qualitative studies (*n* = 13) focused in access to healthful choices from different perspectives: about half of the papers studied aspects of food security, around one quarter focused in understanding the food environment, close to one fifth addressed issues of affordability and food supply, and one study assessed coping strategies for lack of food access.

### Assessment of the quality of research

For quantitative studies, quality was assessed through three dimensions: (i) type of design, (ii) comparison group or not, and (iii) control for potential confounders (i.e. adjusted models). As summarized in Table 3, most studies relied on cross-sectional designs (80%). The rest of the studies were a mix of geospatial analyses (9.1%), cohort and longitudinal studies (9.1%), and only one study was based on a case-control design (1.8%). About 82% of the studies had a comparison group, which was commonly operationalized as urban non-poor populations, rural poor populations, or as comparisons between different subgroups of urban poor population (i.e. differences in income within poor groups, different levels of FI, amongst others). Among studies lacking a comparison group, they were mainly cross-sectional studies [38, 39, 42, 43, 47, 52, 77, 81, 88, 89] that intended to provide descriptions of urban poverty in terms of nutrition outcomes. Close to 70% of all quantitative studies controlled for confounders and presented adjusted models. However, none of the geospatial analyses did so [42, 52, 78, 80, 91], neither the case-control study [48]. By contrast, 75% of the cross-sectional designs [37, 39, 40, 45, 46, 49–51, 54–56, 59, 61–66, 70–77, 82–84, 86, 87, 89, 90] and all the cohort and longitudinal studies controlled for confounders [44, 53, 60, 69, 88].

Among the 13 qualitative studies included in the SLR, all showed adequate research quality (see Table 4). All studies were found to have an adequate theoretical approach with clear aims, and a well-established study design including sample characteristics and qualitative sampling processes. Similarly, all the studies provided a description of the data collection process, recording and transcription of study materials, the study context and participants, and addressed some potential research biases. In terms of data triangulation, which is an important validity aspect of qualitative approaches, most studies reported collecting data through different sources and linking them for purposes of analysis; the only two exceptions were the studies by Dubowitz et al. [97] and Hammelman [99]. Despite their lack of triangulation, both studies were rated as having richness in data. In fact, all studies but one were rated as having dense and rich qualitative data; with the exception of a study focusing on FI among homeless and marginally housed adults in Sydney, Australia [104]. Qualitative studies applied different data collection techniques such as in-depth interviews [92, 95, 96, 98, 99, 101, 103, 104], focus groups [93, 94, 97, 101], participant observation [95, 101], open-ended questionnaires [102] and photovoice [100].

### Content and thematic analysis

Given the diversity of designs, methodological and measurement approaches, instead of summarizing effect sizes or aiming at a meta-analysis, we took a qualitative thematic approach to synthesize and analyze the literature. From such perspective, four broad categories emerged: (i) elements that affect access to healthy eating in individuals in urban poverty, (ii) FI and urban poverty, (iii) risk factors for the nutritional status of urban poor and (iv) coping strategies to limited access to food.

#### Elements of urban poverty that affect access to healthy eating

Urban poverty exerts different pressures which lead, in many cases, to problems of access to a healthy diet that are as serious as in rural areas (Supplementary Table 4). One of the risk factors documented in the literature for this lack of access are the economic barriers faced by the urban poor. These studies provide evidence that healthy diets are expensive, which leads to dose-response socioeconomic inequities in food choices. For example, in urban settings budgetary restrictions in the selection of food can lead to the consumption of diets that are very low in animal protein [51], or may disrupt requirements among populations with special dietary needs [92, 101]. Urban dwellers in the lowest income deciles, allocate a higher proportion of their family income to food consumption [41, 57], and may find restrictions to buying healthy foods [93].

In addition, low income urban neighborhoods, tend to have less access to healthful foods, thus, linking economic constrains of the population and place of living to a magnified lack of access to healthy foods [78]. There are effects of the market structure on access to food in urban poor areas, a common finding was a lower supply of supermarkets [42, 78, 91] that can lead to food deserts. In addition, supermarkets in urban poor areas tend to offer less variety of healthy products (i.e. fresh produce) and oftentimes products of lower quality [71]. Such fragmented market can lead to the establishment of informal arrangements, especially in low- and middle-income countries, such as street traders and house shops that are more likely to be unstable and deregulated [43, 85]. Corner shops are another common source to meet food demand, but this has been associated with increased consumption of ultra-processed foods and inversely associated with home meal preparation, positive beliefs and self-efficacy toward healthy food [55].

Among poor urban dwellers accessing healthier choices commonly requires “out-shopping” defined as shopping outside of your residential area, but this is limited by transportation cost and lack of public transportation access [42]. In addition, this implies additional direct costs (i.e. transportation) and opportunity cost (i.e. time spent) in food purchasing [99]. This can be an even larger barrier to access when experiencing health conditions affecting physical mobility [92].

An additional barrier faced by the urban poor is the *lack of social networks* that allow them to access food during difficult times. Urban studies have documented less reciprocity with food exchanges than those observed in rural areas [68].

#### Food insecurity and urban poverty

An important body of literature emerged documenting the relationship between FI and urban poverty. FI is defined as “the limited or uncertain availability of nutritionally adequate and safe foods; or the limited and uncertain capacity to acquire adequate food in socially acceptable ways” [105]. This literature was grouped into: quantitative studies that address the determinants of FI, quantitative studies that analyze how FI is associated with unfavorable nutrition outcomes among the urban poor, and qualitative studies documenting experiences of FI among urban vulnerable populations.

##### Determinants of FI in poor urban settings

Studies from all regions of the world informed the literature on determinants of FI in poor urban settings. Almost all studies operationalized FI through experience-based scales. Most of the studies were based on cross-sectional designs and logistic regression analysis (see Supplementary Table 5).

One of the main FI risk factors identified in the literature was low household income; among those living on urban and peri-urban areas, low income increased risk of FI [38, 44–46, 50, 53, 58, 59, 65, 72, 76, 82, 84, 89]. Similarly, a study found that lower socioeconomic status and higher levels of unemployment were associated with a higher prevalence of FI [37]. Few studies focused on assets-based measures and FI. A study documented that households with inconsistent access to utilities such as electricity or water, medical care, cooking fuel and cash had a significantly higher prevalence of severe FI [66]. Another study reported that access to a personal vehicle was inversely associated with FI [64].

In addition to experience-based FI scales, one study assessed dietary diversity finding similar associations with socioeconomic status. More specifically it documented that lower income adults in urban areas consumed less varied diets and lower amounts of vitamin C, calcium, iron, riboflavin, and zinc –even when compared with their low-income counterparts in rural areas [75].

##### Association between FI and nutrition outcomes among vulnerable urban groups

Studies that examined the association of FI and nutrition outcomes were mainly from the Americas and Africa, and were based on cross-sectional designs but used different data analysis approaches (see Supplementary Table 6). The literature found that FI is a risk factor for malnutrition of the urban poor. Few studies assessed the association between FI and stunting, and did not reach consensus. While a study documented that in poor urban settlements children under 5 years of age living in FI households were at greater risk of stunting [69], others reported that FI was not significantly associated with stunting among adolescents [62].

Most of the studies assessed the relationship between FI and overweight and obesity leading to mixed findings, partially because study populations were diverse. For example, among schoolchildren living in urban FI households a higher prevalence of overweight was documented [73]. But such associations could not be confirmed among adolescents [56, 61] or preschool children [79, 87]. Similarly, the association also depended on the severity of the FI [67] and the syndemic effect with other factors like parental stress [49, 61].

##### Qualitative approaches to FI in poor urban settings

The *qualitative studies* included in the systematic review were conducted mostly in poor urban areas of high-income countries. Collectively, these studies exemplify the complexity of food access challenges in urban areas and emphasize that food availability is a necessary but not sufficient condition for adequate food access as de facto it depends on other elements as well. Among poor urban older adults living alone with physical and motor limitations, as well as lack of transportation, and social isolation increase the risk of FI [98]. Among the homeless FI was related to insufficient income from government welfare programs, low affordability of fresh food, transportation barriers, lack of safe shelter and housing, and limited food storage capacity [94] [95]. In fact, challenges with access to a kitchen and inadequate spaces to store food emerged in other studies as factors increasing FI [104].

Qualitative studies focusing on mothers living in poverty in urban areas revealed specific food access and healthy eating challenges. In large Metropolitan areas, the major limitations for adequate family nutrition were limited time for food shopping and cooking, as well as finding time for family activities, childcare and difficulties in transportation to and from the food stores [97]. Another factor that emerged is that mothers prioritize food pricing and optimization of food usage when making food selections, oftentimes sacrificing quality [96, 101]. Mothers living in poor urban settlements also referred to an unhealthy food environment in their communities due to the abundance of street vendors and food stores selling junk food [102].

The qualitative studies also documented FI related challenges faced by people who live in urban areas, like increased feelings of anxiety, worry, shame, and uncertainty [103]; and limited self-control for chronic disease, since it prevents access to proper nutrition [92]. Moreover, while social protection and food assistance programs, such as community kitchens, help by providing access to basic nutrition, are insufficient to fully resolve their FI related challenges [104].

#### Risk factors of the nutritional status of the urban poor

Urban poverty poses major challenges for adequate food access and nutrition outcomes among the urban poor, exposing them to nutritional risks with long-term consequences. Our systematic review identified associations between food access barriers and increased risk for poor nutrition outcomes through three different pathways. First, urban poor have an increased risk of consuming unhealthy and energy dense foods associated with a higher prevalence of overweight and obesity [47, 86]. Second, urban poverty was found to increase the chances of chronic undernutrition, leading to higher obesity prevalence in future stages of life [88]. And third, the review suggested that psycho-social factors are important determinants of obesity through plausible biological links with stress and feelings of despair commonly experienced by people living in urban poverty [49, 76, 104].

#### Coping strategies for limited food access

An aspect that emerged from the literature refers to strategies used by the urban poor to obtain food and, among them, the use of food banks [68, 92, 98] and community kitchens [92] stand out. These studies found that beneficiaries considered such support strategies valuable but insufficient to fully mitigate hunger and lack of access to food, hence, families and individuals need other coping mechanisms like selling food on the streets to generate income, while at the same time have more access to food [54]. Other strategies implied skipping meals or eating smaller portions [103, 104]. These unhealthy coping mechanisms were more prevalent among mothers, who buffer their children against FI [53, 103]. Finally, other strategies included buying stolen food at a lower price or eating food from garbage [104].

### Conceptual framework

**Fig. 3:**
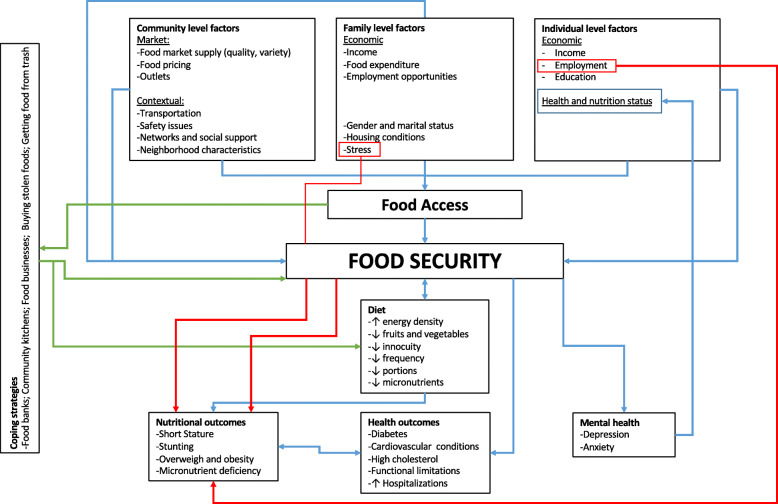
Conceptual Framework of nutrition and urban poverty

Figure 3 presents a conceptual framework that intends to graphically depict the key themes that emerged from our literature review. At the center two key themes shape the relationship between nutrition and urban poverty: access to food and household food security status. These elements are determined by the factors summarized in the left part of the Figure, which are grouped in different ecological levels: community, family and the individual. These themes and factors help explain nutritional and health outcomes in the context of urban poverty including overweight and obesity, short stature and stunting. The conceptual framework also highlights the coping strategies used among the urban poor to deal with food access challenges as well as FI.

## Discussion

According to previous studies, in general, urban diets are likely to be more varied than rural diets [10]. However, this urban advantage strongly diminishes as a function of socioeconomic status representing a major social and health inequity in urban setting. In cities, food, for the most part, is bought and not grown for consumption. This implies that their access to healthy foods is strongly linked to income and to the structure of the food system, including its corresponding supply and access chains; i.e., “from farm to table”. These factors are two key determinants of the type of effective policies needed for urban populations to have access to a healthy diet [51, 57].

The systematic literature review confirms that these determinants of food access in urban areas emerge in the context of poverty and high levels of FI of different countries [37, 44–46, 65, 84], which are highly prevalent of poor nutrition and health outcomes [39, 69, 73, 76]. Empirical evidence indeed supports the existence of a socioeconomic gradient in access to healthy food in urban areas [51, 92]. The review emphasizes that access to food in urban areas is a complex process with multiple determinants and that it cannot be assumed that this access is always better for populations in urban vs. rural areas.

An important structural economic challenge for food access among the socioeconomically disadvantaged in urban areas is that the prices of healthy foods can be higher in poor neighborhoods, which at the same time also tend to have fewer food retail stores [41, 42]. This is a strong structural barrier for families living in urban poverty. The structural challenges surrounding the food supply systems and markets in vulnerable urban areas means that sometimes individuals need to travel to other places to access healthy food, which increases costs (i.e. transportation) and mental stress due to the physical barriers to access food in their own communities. This adverse situation for the urban poor is compounded by problems of poor transport infrastructure as well as high community crime rates [42].

An interesting phenomenon that emerged from the literature –that in future studies may help compare challenges to food access among the urban and rural poor– is related to the nature of the social fabric and networks. Specifically, studies found that because urban networks tend to be weaker and, in the case of coping with FI, it may prevent families from “borrowing” or exchanging food with others [68, 98].

Our review also found that urban poverty leads to increased risk of poor nutrition outcomes including stunting, overweight and obesity. Three themes that may help explain this finding emerged. First, the evidence indicates that urban environments foster a greater consumption of ultra-processed foods with high content of calories, fats, salt and sugars and very low nutritional value [47, 86]. Likewise, studies show that lack of food-access may lead to skipping meals [53, 103, 104]. This is of public health concern, as it is known that prolonged fasting may predispose to unfavorable metabolic responses [106, 107]. Finally, several articles pointed out how these experiences may be leading to mental health problems as a result of shame, and despair among those affected by FI without the ability to properly cope with it [76, 104]. FI- related mental health stressors in turn can also increase the risk of cardiometabolic alterations and nutritional status [108–110]. Previous studies have established a strong plausibility for linking mental stress with the risk of overweight and obesity, mainly due to the increased release of hormones and neurotransmitters that can cause an increase in visceral adiposity and changes in the areas of the brain where hunger and satiety are regulated [108–110].

A substantive body of FI literature was identified. It is clear that FI in urban areas is strongly driven by income limitations. Specifically, low-income households need to allocate a high proportion of their total expenditure to food and are extremely vulnerable to any external shock including unemployment, health problems and food price inflation [45, 46, 65, 84]. Similarly, the literature documented that the impact of FI on poor health is compounded by the fact that low-income urban households tend to have poor sanitation and other essential housing infrastructure and goods [46].

Given the findings from this review, it is not surprising that FI among the urban poor [49, 73, 76] has been associated with poor nutrition outcomes. This highlights the relevance of monitoring FI in urban populations. Food insecurity experience scales (FIES) are important in capturing this phenomenon among the urban poor, and efforts should be made to capture the different severity levels (i.e. mild, moderate, severe).

Another theme of great relevance is that social protection and food assistance programs designed to facilitate food access - such as monetary or in-kind transfer schemes, community kitchens and food banks - are insufficient by themselves to fully resolve the FI problem because they do not address barriers such as lack of cooking facilities or food storage, and competing health or housing expenses. Therefore is not surprising that socially unacceptable coping strategies, such as taking food from garbage, were reported, illustrating the depth of the negative effects of urban poverty on the right to food [104]. Interestingly, these FI coping behaviors contrast with those observed in rural areas, such as food exchanges and small family agriculture for self-consumption [44, 68].

Urban poverty poses unique and diverse challenges and pathways to food access and the ability of families to consume healthy and nutritious diets that prevent access to healthy diets. It is possible that the nature of cities including unplanned built environments and challenging social network structures prevent low income individuals from finding strategies to cope with FI and lead to socially unacceptable behaviors to access foods.

In terms of the quality of the research examined, from a quantitative standpoint, most studies relied on cross-sectional designs, which do not allow to draw causal inferences, therefore there is a literature gap that requires further research with a longitudinal approach. While in the future more robust designs would be desirable, it should also be stressed that literature using different samples and conducted in a diverse set of countries is yielding similar conclusions in terms of the food access challenges and poor nutrition outcomes among the urban poor. However, further research needs to be conducted with more explicit comparison groups (such as urban population in very small, small, medium size cities, and metropolis) to answer the following questions: i) What is the role of social protection in terms of reducing FI for the vulnerable population? ii) Should it be continuous for some groups and intermittent for others? iii) What interventions should be put in place when food prices rise or economic conditions worsen to make sure the vulnerable are protected? iv) Should economic sanctions or incentives be put in place to induce away the demand of processed food consumption? v) What channels are more effective to assure quality access to food for the poor in urban settings? Finally, vi) What combination of policies could be recommended to be exerted together rather than in isolation?

Ideally, the proposed framework that emerge from the literature review should aid in the development of future research addressing food insecurity and nutrition outcomes in the context of urban poverty.

Furthermore, the operationalization of the definitions of “urban” and “poverty” were highly heterogenous across studies, hence, limiting the comparability of their findings. Future studies are needed to better harmonized definitions of poverty and the urban space, preferably studies should stratify samples according to the urban population size. The quality of qualitative studies was high overall, although there is room for improvement in terms of triangulation and reporting more explicit details on how data were retrieved, coded and analyzed.

In addition to the lack of uniform high quality across studies, this review has other important limitations when interpreting its findings. First, search algorithms were limited to specific nutrition outcomes that, despite being the more salient ones, might have excluded studies addressing other outcomes. Second, although FI is strongly linked to poverty, it is possible that some relevant studies that did not mention the word “poverty” but are related to disadvantages or inequalities, may have been left out from the review. Third, the review only included studies published in Spanish or English which may have led to excluding relevant literature published in other languages. Fourth, the search engines used retrieved studies in published academic journals, therefore the review may have excluded relevant studies only published in the grey literature. Fifth, the review did not conduct a meta-analysis to understand effect sizes of associations. This was not possible due to the strong heterogeneity across studies including the many different ways in which “poverty” and “urban” were defined. However, in recognition of such limitation, we performed a qualitative thematic analysis of the selected studies. Perhaps future reviews could narrow the search strategy to only studies that are more homogenous with regards to operational definitions of exposures and outcomes. Sixth, it is also important to note that mixed methods studies were excluded from the analysis due to the complexity of their systematization.

## Conclusions

The systematic literature review evidenced the intricate link between urban poverty, food access, household food security, and nutrition. A contribution of this review is that it identified distinct barriers present in urban areas, questioned the supposedly “urban advantage” regarding access to healthful food, and developed a conceptual framework that focuses on the particular difficulties to achieve household food security among the urban poor through improved food access, which should inform future research. This systematic review provides consistent evidence that the right to food among those living in urban poverty is compromised; this is particularly worrisome considering that an urban setting is where the majority of the countries’ populations now live or will be living in the near future. It is essential that the social and public health sectors engage in addressing these issues jointly due to the complexity highlighted by the framework developed based on the available scientific evidence.

## Supplementary Information

**Additional file 1.**

## Data Availability

Not applicable
