# Supplementary material for: Urban poverty and nutrition challenges associated with accessibility to a healthy diet: a global systematic literature review
Source: Int J Equity Health. 2021 Jan 20;20:40. doi: 10.1186/s12939-020-01330-0 (PMC7816472; doi:10.1186/s12939-020-01330-0)
Supplement: Supplementary file 1 — Additional file 1. [file 12939_2020_1330_MOESM1_ESM.docx]

**Supplementary Table 1. Boolean search system for each research question**

| **Research question** |  | **Search system** |
| --- | --- | --- |
| How urban poverty affects the access to a healthy diet |  | (urban OR semiurban OR semi-urban OR periurban OR “inner city” OR metropolis OR metropolitan OR city) AND (poor OR poverty OR vulnerability OR socioeconomic status “OR” socioeconomic level “OR” low income”) AND (“correct diet “OR” good diet “OR” proper diet “OR” healthy diet “OR” food security “OR” food insecurity “OR” food safety “OR” varied diet “OR” balanced diet “) AND (access) |
| What aspects of urban poverty lead to alterations of the nutritional status |  | (urban OR semiurban OR semi-urban OR periurban OR “inner city” OR metropolis OR metropolitan OR city) AND (poor OR poverty OR vulnerability OR “socioeconomic status” OR “socioeconomic level” OR “low income”) AND (anemia OR obesity OR overweight OR “micronutrient deficiency” OR “micronutrient malnutrition”) |

**Supplementary Table 2. Quality assessment guidelines for quantitative studies**

| **Attribute** | **Description** | **Elements assessed** |
| --- | --- | --- |
| Type of design | -Assess if the design is experimental, quasi-experimental, or non-experimental  -Determine of it’s a longitudinal (i.e. prospective or retrospective) or cross-sectional design | -Can causality be ascertained?  -Can association be ascertained? |
| Operationalization of exposure | -Assess if the urban space is defined  -Identify how are poor or vulnerable populations defined  -Assess if comparison groups are defined | -Is there an explicit definition of the urban space? Is it credible given international standards?  -How is poverty or vulnerability described?  -Are specific non-urban or non-poor comparisons addressed? |
| Outcome variables | -Assess if the article addresses access issues to healthful foods and/or nutritional outcomes of interest  -Identify how are access issues and/or nutritional outcomes operationalized | -Are access and/or nutritional outcomes credibly measured?  -Are they correctly addressed for the type of context or population? |
| Controls for confounding | -Assess if key confounding variables are controlled in the study  -Determine if the statistical technique for controlling confounders is appropriate | -Are key confounding variables identified?  -Are confounding variables correctly operationalized?  -Are the statistical techniques for controlling confounders adequate? |

Note: Adapted from Khan (2003)

**Supplementary Table 3. Quality assessment guidelines for qualitative studies**

| **Area** | **Key questions** | **Elements assessed** |
| --- | --- | --- |
| Theoretical approach (underlying theory and principles applied to the research) | -Is a qualitative approach appropriate?  -Is the study clear in what it seeks to do? | -Research investigates phenomena that are not easy to quantify or measure accurately  -What the study is investigating is set out early and clearly. |
| Study design (robustness of the design of the research) | -How defensible/rigorous is the research design/methodology? | -Research design captures appropriate data and has an appropriate plan of analysis.  -Sample and sampling method are described. |
| Data collection (description of data collection methods) | -How well was data collection carried out? | -There is a definition of how data was collected, recorded and transcribed  -There is an assessment of appropriateness of data collection given the aims of the research |
| Validity (discussing the reflexive position of the researcher, the context in which the research was conducted and the reliability of the actual data) | -Is the context clearly described?  -Were the methods reliable? | -Describes the context of the research in terms of the physical context, the participants, and any potential context bias considered by the authors.  -Defines why the data collection methods are appropriate for the research question (ideally, more than one method should have been used to collect data). |
| Analysis (description of the qualitative methods for systematization and analysis) | -Are the data “rich”?  -Is the analysis reliable?  -Are the findings convincing?  -Are conclusions adequate? | -Describes the “richness” of the data (i.e. in-depth, convincing, compelling and detailed).  -Describes how was data coded, and if there was a process for consensus and checks.  -Findings are presented clearly and logically.  -Extracts from original data are included. |

Note: Adapted from NICE Methodology checklist for qualitative studies (2012)

**Supplementary Table 4. Synthesis of studies that report barriers in access to food among urban poor**

| Author (year) | Region  (population) | Type of study (design) | Variable | Barriers |
| --- | --- | --- | --- | --- |
| Barosh et al. (2014) | Sidney, Australia  (Food retail stores) | Cross-sectional  (Descriptive) | Food basket affordability | ↓Income in disadvantaged neighborhoods: ↑food expenditure  (C, E) |
| Battersby & Peyton (2014) | Cape Town, South Africa  (Food retail stores) | Geospatial | Distribution of food retail stores | ↓Income neighborhoods: ↓Supply of supermarkets, fresh food supply, ↓infrastructure of supermarkets (M, C) |
| Battersby (2019) | Multi-site (Households) | Geospatial | Food security | ↓Income neighborhoods: ↑food procurement in local stores (M) |
| Chan et al. (2015) | Toronto, Canada  (Adults with diabetes) | Qualitative  (Thematic analysis) |  | Budget constraints affect food selection (E). Medical condition affects physical possibilities of access to food (P) |
| Cotter et al. (2017) | Washington, USA  (Adults) | Qualitative  (Thematic analysis) |  | The cost at the farmers’ markets (E) |
| Cunha et al. (2011) | Rio de Janeiro, Brazil  (Adults 19 to 65 years old) | Cross-sectional  (X^2^) | Dietary pattern | ↑Budget constraints: ↓selection of foods rich in protein of animal origin (E) |
| Davies et al (2017) | Brazil | Geospatial | Food retail stores | Access to healthy food was not linked to neighborhood poverty.  ↑Poorer areas: ↓prices (E, M) |
| Garcia et al. (2018) | Baltimore, USA (Adult caregiver of 4 to 19-year-old children) | Cross-sectional  (X^2^) | Dietary pattern | ↑Purchase of food from corner stores, fast food restaurants, and convenience stores: ↑Fast Food Consumption: (M) |
| Hammelman (2018) | Washington, USA (Migrant women) | Qualitative  (Thematic analysis) |  | Expensive transportation (C) |
| Jones & Charlton (2015) | Port Vila, Republic of Vanuatu  (Household) | Cross-sectional  (ANOVA) | Fruit and vegetable affordability | ↓income: ↓% spending on fruits and vegetables (E) |
| Levay et al. (2013) | Dhaka, Bangladesh (Pregnant women and new mothers) | Qualitative  (Thematic analysis) |  | Food prices at the market (M) |
| Leung et al. (2016) | New York City, USA (Youth 11 to 14 years old) | Qualitative  (Thematic analysis) |  | Unhealthy foods were cheaper and heavily advertised (M) |
| Morton et al. (2008) | USA  (Adults) | Cross-sectional  (ANOVA) | Coping strategies for food access | Urban low-income group: ↓food “borrowing” or exchanging  (C) |
| Odunitan-Wayas et al. (2018) | Cape Town, South Africa (Supermarket costumers) | Cross-sectional  (X^2^) | Purchase pattern; access to food retail stores;  food environment perception | ↓Income: Fruits and vegetables are perceived as of low quality. (M) |
| Park et al. (2011) | New York City, USA (Female caregivers) | Cross-sectional (Generalized estimating equation) | Dietary patterns | ↑Neighborhood poverty and ↑fast-food restaurants: ↓adherence to the healthy dietary pattern (E,M) |
| Russell & Heidkamp (2011) | New Haven, USA (Food retail stores) | Geospatial | Distribution of food retail stores | ↓Income, ↑poverty rates and ↓vehicle access: ↓availability of supermarkets (E,C,M) |
| Wagner et al. (2019) | Multi-site | Cross-sectional  (Spearman’s Rho correlation) | Dietary pattern; household food sources | ↓Household supermarket access: ↑FI (M) |
| Zhang & Debarchana (2016) | Hartford, USA (Food retail stores) | Geospatial | Distribution of food retail stores | ↓Availability of supermarkets: ↓healthy food access (M) |

Notes: (E): economic barrier; (M): market barrier; (C): contextual/geographic barriers; (P): physical conditions; FI: Food insecurity

**Supplementary Table 5. Synthesis of studies looking at determinants of food insecurity in urban areas**

| Author (year) | Region  (population) | Type of study (design) | Variable | SBE | Results |
| --- | --- | --- | --- | --- | --- |
| Agarwal et al. (2009) | India, Delhi  (Households) | Cross-sectional  (Logit) | Food insecurity | Yes | ↑FI, ↑Unemployment  ↑FI, ↓SES |
| Akinboade & Adeyefa (2018) | Tshwane , South Africa (Households) | Cross-sectional  (ANOVA) | Food insecurity | Yes | ↑FI, ↓Income |
| Belachew et al. (2012) | Jimma, Ethiopia  (Adolescents 13 to 17 years old) | Longitudinal  (Logit) | Food insecurity | Yes | Urban FI> peri-urban> rural  ↑Chronic FI (urban), ↓Income |
| Birhane et al. (2014) | Addis Ababa, Ethiopia  (Households) | Cross-sectional  (Logit) | Food insecurity | Yes | ↑FI, ↓Income |
| Costa et al. (2019) | Belo Horizonte, Brazil  (Restaurant workers) | Cross-sectional  (Logit) | Food insecurity | Yes | ↑FI, ↓Income |
| de Souza Bittencourt et al. (2013) | Salvador, Brazil  (Children 6 to 12 years old) | Cross-sectional  (Multinomial Logit) | Food insecurity | Yes | ↑FI, ↓Income  ↑FI, ↓Housing conditions  ↑FI, Female head of the household  ↑FI, ↑Household size |
| Faye et al. (2011) | Nairobi, Kenya  (Households) | Longitudinal/  Panel  (X^2^) | Food insecurity | Yes | ↑FI, ↓Income |
| Kasper et al. (2000) | USA  (Legal immigrants) | Cross-sectional  (Logit) | Food insecurity | Yes | ↑FI, ↓Income  ↑FI, receipt of food stamps  ↑FI, Latino ethnicity ↑FI, poor English |
| Kirkpatrick & Tarasuk (2011) | Toronto, Canada  (Households) | Cross-sectional  (Logit) | Food insecurity | Yes | ↑FI, ↓Income |
| Martinez et al. (2019) | Baltimore, USA  (Households) | Cross-sectional  (Logit) | Food insecurity |  | ↓FI, access to a personal vehicle |
| Martin-Fernandez et al. (2013) | Paris, France  (Adults) | Cross-sectional  (Logit) | Food insecurity | Yes | ↑FI, ↓Income  ↑FI, Female head of the household  ↑FI, single-parent homes |
| McCordic & Abrahamo (2019) | Mozambique  (Households) | Cross-sectional  (X^2^ and Fisher’s) | Food insecurity | Yes | ↑FI, inconsistent access to water, electricity, medical care, cooking fuel, and cash |
| Omidvar et al. (2013) | Iran  (Migrant women) | Cross-sectional  (Logit) | Food insecurity | Yes | ↑FI, female-headed households, head and spouse ↓level of education, Sunni sect, and illegal residential status, unemployment, ↓SES |
| Ramsey et al. (2012) | Brisbane, Australia  (Adults 25 to 45 years old) | Cross-sectional  (X^2^) | Food insecurity | Yes | ↑FI, ↓Income |
| Vedovato et al. (2016) | Baltimore, USA  (Households) | Cross-sectional  (X^2^) | Food insecurity | Yes | ↑FI, ↓Income  ↑FI unemployed |
| Vuong et al. (2015) | Ho Chi Minh, Vietnam  (Household) | Cross-sectional  (Logit) | Food insecurity | Yes | ↑FI, ↓Income  ↑FI, ↓Consumption of fruits and vegetables |
| Yaemsiri et al.(2012) | New York City, United States  (Adults) | Cross-sectional  (t test) | Food insecurity | No | ↑FI, foreign born  ↑FI, ↓education  ↑FI, ↓income  ↑FI, ↑number of children |

Notes: FI: Food insecurity; SES: Socioeconomic status; Logit: Logistic regression analysis

**Supplementary Table 6. Synthesis of studies assessing how food insecurity in urban areas affects nutritional outcomes**

| Author (year) | Region  (population) | Type of study (design) | Variable in health or nutrition | Results |
| --- | --- | --- | --- | --- |
| Chambers et al. (2009) | Multi-site, USA (Dyad Mother-Child) | Cross-sectional  (Logit) | BMI ^a^ | ↑Obesity mothers, ↑Unstable home (FI, income, stress) |
| Gundersen et al. (2008) | Multi-site, USA  (Children 10 to 15 years old) | Cross-sectional  (Logit) | Overweight, risk of overweight ^b^ | FI without significant effect for overweight or risk of overweight |
| Lohman et al. (2009) | Multi-site, USA (Dyad caregiver- adolescents 10 to 15 years old) | Cross-sectional  (Logit) | Overweight, obesity ^c^ | FI without significant effect for overweight/obesity in adolescents.  ↑Overweight/obesity in adolescents, FI-Caregiver stressors interaction |
| Lopes et al. (2013) | Rio de Janeiro, Brazil (Adolescents 12 to 18 years old) | Cross-sectional  (Pearson-X^2^ test) | Overweight, stunting ^d^ | FI without significant effect for overweight and stunting |
| Miller et al. (2008) | Chelsea, MA, USA (Households with children) | Cross-sectional (X^2^, ANOVA) | Overweight, obesity, underweight, anemia, high lead ^e^ | ↑Obesity, ↑FI  FI without significant effect for overweight, underweight, anemia and high lead |
| Mutisya et al. (2015) | Nairobi, Kenya  (Dyad mother-Child 6 to 24 months old) | Cohort  (Cox reg., Kaplan-Meier Curves) | Stunting ^f^ | ↑Stunting, ↑FI in moderate poverty households |
| Ortiz-Hernández et al. (2007) | Mexico City  (Elementary students 4° to 6° grade) | Cross-sectional  (Logit) | Overweight ^g^ | ↑Overweight, ↑FI |
| Villamor et al. (2017) | Multi-site, 9 Latin-American countries (Households) | Cross-sectional (Poisson reg., linear reg.) | Metabolic syndrome ^h^ | ↑Metabolic syndrome, ↓FI in men  FI without significant effect for metabolic syndrome in women and children |
| Whitaker & Orzol (2006) | Multi-site, USA (3-year-old children) | Cross-sectional  (Cochran-Armitage trend test, Logit) | Obesity ^i^ | ↑Obesity, ↓FI in African American children  FI without significant effect for obesity prevalence in Caucasian and Hispanic children, nor overall sample |

Notes: FI: Food insecurity; BMI: Body mass index; Logit: Logistic regression analysis

^a^ Mothers obesity: BMI ≥30kg/m2; overweight children: age- and sex- specific BMI z-score >95th percentile (CDC growth charts).

^b^ Two classifications: A) age- and sex-specific reference values of the CDC growth charts: 1) underweight (BMI < 5th percentile); 2) normal weight (BMI > 5th and < 85th percentiles); 3) at risk for overweight (BMI ≥ 85th and, <95th percentiles); and 4) overweight (BMI ≥ 95th percentile). B) age- and sex-specific reference values developed by the IOTF.

^c^ Two classifications: A) age- and sex-specific reference values of the CDC growth charts. B) percentiles by the IOTF.

^d^ Weight status: z-score cut-offs proposed by the WHO: underweight (<-2), normal weight (≥ -2 and ≤ +1) and overweight (>+1). Height-for-age index z-score <-2 were classified as stunting.

^e^ Overweight: Height/weight scores above 85^th^ percentile; Obese: above 95^th^ percentile, and Underweight: below 5^th^ percentile. Anemia: hematocrit counts below 32

^f^ z-scores for the Height for Age using the ‘WHO Child Growth Charts and WHO Reference 2007 Charts’ for children aged up to 2 years.

^g^ Overweight children: age- and sex- specific BMI z-score >95^th^ percentile (CDC growth charts). According to the National Cholesterol Education

^h^ Program’s ATP III criteria (Abdominal obesity was defined as WC>102 cm in men or WC >88 cm in women; high fasting glucose as ≥100 mg/dl; high blood pressure as systolic blood pressure ≥130 mmHg, diastolic blood pressure ≥85 mmHg or treatment with an antihypertensive drug; low HDL cholesterol as serum concentration).

^i^ BMI percentiles for age and sex using the Centers for Disease Control and Prevention 2000 growth reference
